# Intestinal RICT-1 regulates the larval germline progenitor pool via the vitellogenin VIT-3 in *C. elegans*

**DOI:** 10.1101/2025.01.08.632040

**Published:** 2025-01-09

**Authors:** Anke Kloock, E. Jane Albert Hubbard

**Affiliations:** Department of Cell Biology, NYU Grossman School of Medicine, New York, NY 10016

**Keywords:** TORC2, Stem Cells, Rictor, Vitellogenins

## Abstract

Populations of proliferating cells such as stem cells and tumors are often nutrient responsive. Highly conserved signaling pathways communicate information about the surrounding environmental, organismal, and cellular nutrient conditions. One such pathway is the Target of Rapamycin (TOR) pathway. The TOR kinase exists in two complexes, TOR complex 1 (TORC1) and TOR complex 2 (TORC2). TORC1 has been researched extensively and its regulation, particularly by amino acids, is well characterized. TORC1 activity promotes both stem cell fate and proliferation in the *Caenorhabditis elegans* hermaphrodite germline stem cell system to facilitate expansion of the larval germline Progenitor Zone (PZ) pool in response to nutrients. By contrast, a role for TORC2 in germline development has not been investigated. Here, we show that RICT-1, the sole ortholog of the TORC2-specific component RICTOR, also promotes expansion of the larval PZ, acting largely through SGK-1. Further, unlike the germline-autonomous role for TORC1 components, intestinal *rict-1* is both necessary and sufficient for full germline PZ pool establishment. Furthermore, neither DAF-2/IIS nor DAF-7/TGF-ß pathways mediate the effects of RICT-1. Rather, intestinal RICT-1 likely acts via vitellogenins, intestinally produced yolk proteins previously characterized for provisioning the adult germ line, but not previously characterized for a role in larval germ line development. By comparative RNA-seq on staged L4 larvae, we found vitellogenin genes among highly differentially abundant mRNAs. Genetic analysis supports a role for *vit-3* in germline development in a linear pathway with *rict-1*. Our results establish the *C. elegans* germ line as a fruitful model for investigating TORC2 and its connection to stem cells and lipid biology.

## INTRODUCTION

Highly conserved signaling pathways govern cellular responses to nutrients and metabolites. These responses are required for the proper regulation of pools of proliferating cells such as stem cells and tumor cells (Puca et al., 2022; Tu et al., 2023).

TOR is a highly conserved serine/threonine kinase that is conserved from yeast to mammals and integrates nutritional signals to regulate cellular responses (reviewed in (Liu and Sabatini, 2020)). TOR acts in two complexes bearing key subunits Raptor in TORC1 and Rictor in TORC2. Both complexes respond to nutritional signals and regulate many nutrient-dependent aspects of stem cell biology (reviewed in (Russell et al., 2011; Saba et al., 2021)). Albeit differently, both complexes respond to amino acid availability and impact lipid metabolism (reviewed in (Caron et al., 2015; Szwed et al., 2021; Zoncu et al., 2011)) and homeostasis (Kim and Chen, 2004). TORC2 furthermore promotes *de novo* fatty acid and lipid synthesis in mice (Guri et al., 2017) and worms (Jones et al., 2009; Soukas et al., 2009). In *C. elegans*, our prior work established that TORC1 components and/or downstream effectors affect germline stem cell proliferation and differentiation germ line-autonomously and in response to amino acid provision (Korta et al., 2012; Roy et al., 2018; Roy et al., 2016).

To address the functions of TORC2 distinct from those of TORC1, Rictor mutants have been characterized in rodents, worms, and flies. In all three systems, reducing Rictor activity results in animals that are smaller and develop slower (Guertin et al., 2006; Hietakangas and Cohen, 2007; Jones et al., 2009; Soukas et al., 2009). Mutants in rodents and worms display high fat accumulation (Frei et al., 2023; Jones et al., 2009; Soukas et al., 2009; Yen et al., 2010), suggesting Rictor facilitates trafficking of lipids from the intestine to other organs (Jones et al., 2009; Soukas et al., 2009). Adult *C. elegans* hermaphrodites bearing reduction-of-function mutations in *rict-1*, the sole Rictor ortholog(Jones et al. 2009), exhibit reduced intestinal expression of the vitellogenin gene *vit-3* (Dowen et al., 2016), and produce fewer offspring (Jones et al., 2009; Soukas et al., 2009). While many cellular mechanisms regulate brood size, one is the number of cells in the early adult progenitor zone (PZ) (Agarwal et al., 2018; Kocsisova et al., 2019), the pool of stem and progenitor cells in the distal part of the hermaphrodite gonad (reviewed in(Hubbard and Schedl, 2019)).

Our results show that *rict-1* mutant adults have a reduced brood size and half the normal pool of cells in the PZ. As in other phenotypic contexts, *rict-1* acts via *sgk-1*, but does not act via downstream IIS or TGFß pathways that are implicated in *rict-1* control of dauer entry (O'Donnell et al., 2018) and in larval PZ expansion (Dalfo et al., 2012; Michaelson et al., 2010; Pekar et al., 2017). We show that intestinal RICT-1 is sufficient and necessary for full establishment of the adult PZ. Our results also indicate that during the L4 larval stage, *rict-1* regulates expression of several members of the conserved ApolipoproteinB-like vitellogenins (Perez and Lehner, 2019), and that *vit-3* contributes to expansion of the progenitor pool, likely downstream of *rict-1*.

## RESULTS

### RICT-1 is required for normal germline development

**Figure 1:**
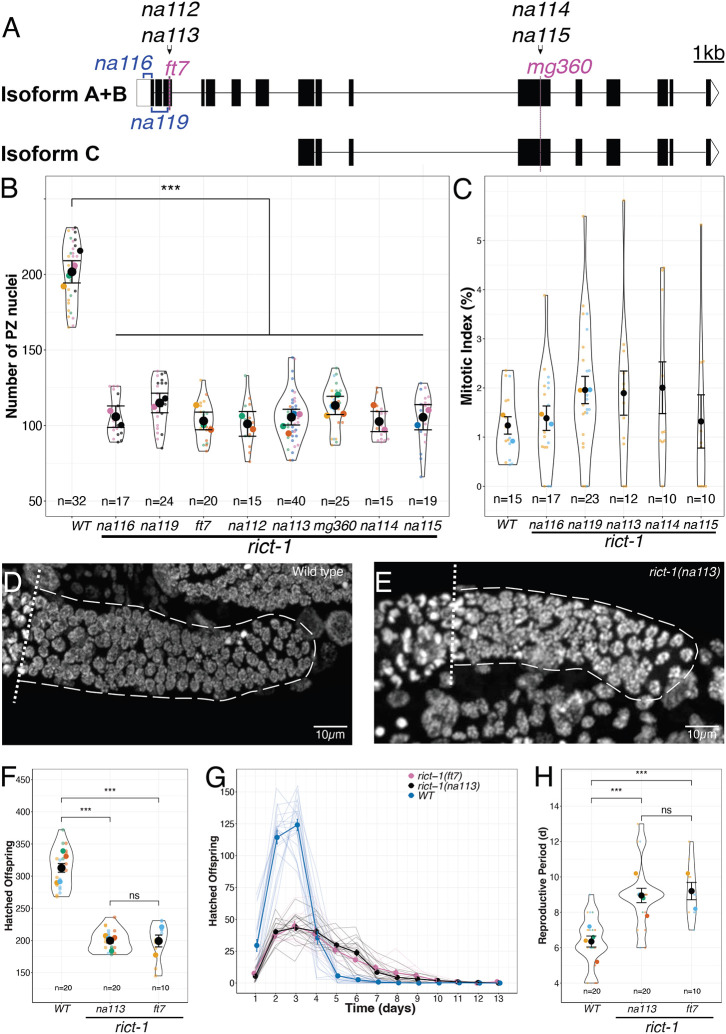
*rict-1* regulates reproduction and the germline progenitor pool. (A) Overview of the eight different *rict-1* mutants used in this study (two pre-existing SNP mutations in magenta, four Stop-in mutants in black, and two knock-out mutants in blue). Scale bar indicates 1kb. Only alleles *mg360*, *na114*, and *na115* affect all isoforms. (B) Number of PZ nuclei for wild type and eight tested *rict-1* mutants. (C) Mitotic index for wild type and five tested *rict-1* mutants in comparison to wild type. (D, E) Micrographs of representative wild type (D) and *rict-1(na113)* mutant (E) progenitor zones. (F) Average of hatched offspring per adult over a lifetime. (G) Hatched offspring per adult over time; the thick lines indicate the average per strain, while the thinner lines indicate single worms. Error bars indicate standard error of the mean. (H) Reproductive period in days. (B, C, F, H) Black circles indicate the means across independent replicate experiments (shown in different colors), while the smallest circles indicate gonad arms (n = number of gonad arms scored). The violin displays the overall distribution of data points. Error bars indicate the standard error of the mean. Statistical tests are summarized in Table S2. The asterisks indicate *** p<0.001, ** p<0.01, * p<0.05 of a Linear Mixed Effects Model with a Tukey Post Hoc Test. Panel B, F, and H were analyzed with a Linear Mixed Model, panel C was analyzed with a Linear Model, and panel G was analyzed with a Generalized Linear Model, all followed up with a Tukey Post Hoc test where appropriate.

To explore effects of TORC2 on the germ line, we examined the effects of reduced *rict-1*. We assessed phenotypes in two available *rict-1* mutants (*ft7* in the third exon and *mg360* in the 11^th^ exon (Jones et al., 2009; Soukas et al., 2009)) and in 6 additional alleles that we generated using CRISPR-Cas9 genome editing. Among three different isoforms (isoform A and B encode identical proteins), *ft7* only affects isoforms A+B, while *mg360* affects all three isoforms (Figure 1A). We generated four CRISPR mutants with Stop-In cassettes (Wang et al., 2018) inserted in the regions of each of the SNPs: *na112* and *na113* in the third exon, and *na11*4 and *na115* in the 11^th^ exon. We generated two additional mutants: one that deletes 241bp of the 5’UTR and 29bp of the first exon (*na116*), and one that deletes 520bp in total across the first three exons (*na119*). We used this strategy so as not to interfere with the PQN-32 locus that resides within the intron between exons 10 and 11. Strains bearing all these mutations, as well as others used in this study are listed in Table S1.

To gauge the effects of *rict-1* on larval accumulation of the stem/progenitor pool, we counted the number of nuclei per distal gonad arm in the distal progenitor zone (PZ) at the adult molt. We chose to assess the progenitor pool at the molt as this represents a developmental timepoint independent of growth rate and at the start of the early adult steady-state (reviewed in(Hubbard and Schedl, 2019)). All eight *rict-1* mutants displayed a similar significant reduction in the number of PZ nuclei, only reaching half of the wild-type level at the L4 to adult molt (average 107 ± 15.4 across all eight alleles versus 202 ± 20.4 in the wild type) (Figure 1B). We further determined the mitotic index and found that, in contrast to *rsks-1* S6 kinase mutants (Korta et al., 2012), *rict-1* mutants display the same mitotic index as wild-type worms (p=0.4) (Figure 1C). Representative images of the wild-type PZ and the *rict-1(na113)* mutant are shown in Figures 1D and 1E, respectively. We do not see differences when comparing anterior versus posterior position gonad arms (Figure S1B). Given that all *rict-1* mutants behaved similarly, our subsequent studies focused on the *na113* allele.

The brood size in mated worms is correlated with the number of PZ nuclei (Agarwal et al., 2018; Kocsisova et al., 2019). To determine whether the PZ pool reduction we observed in the *rict-1* mutants is associated with reproductive defects, we assessed live broods and, consistent with previous results (Jones et al., 2009; Soukas et al., 2009), we observed a significantly smaller number of hatched offspring for both *rict-1* alleles tested (199 ± 9.21 for *rict-1(ft7)* and 200 ± 3.82 for *rict-1(na113)*) compared with the wild type (312.8 ± 6.77)(Figure 1F,G). We also observed that *rict-1* mutants display an extended reproductive period of ~9 days compared with ~6 days in the wild type (8.95 ± 0.41 days for *na113*, 9.2 ± 0.49 for *ft7*, and 6.35 ± 1.39 for the wild type; Figure 1G,H). Since hermaphrodite brood size is sperm limited (Hodgkin and Barnes, 1991), and the correlation with the PZ can only be assessed in the presence of replete sperm, we assessed live broods in mated worms. We found that, unlike the wild type, where male sperm provision significantly increased offspring number, the number of hatched offspring is not increased in mated *rict-1* mutants (Figure S1A). Furthermore, *rict-1* mutants appear more susceptible to damage inflicted by mating, as the mated hermaphrodite *rict-1* mutant survival was negatively impacted by mating, compared with unmated *rict-1* mutants (data not shown). Despite the extended reproductive period, the overall sum of hatched brood of *rict-1* mutants is still reduced in comparison to wild type (Figure 1F). We conclude that RICT-1 promotes normal germline development, and we focused on the PZ pool as this is a primary early aspect of germline development.

### SGK-1 acts downstream of RICT-1 to establish the early adult PZ pool

We next assessed known pathway components that downstream of RICT-1 and RICTOR. Canonically, Rictor phosphorylates SGK (reviewed in (Blackwell et al., 2019; Jang et al., 2022)).

**Figure 2:**
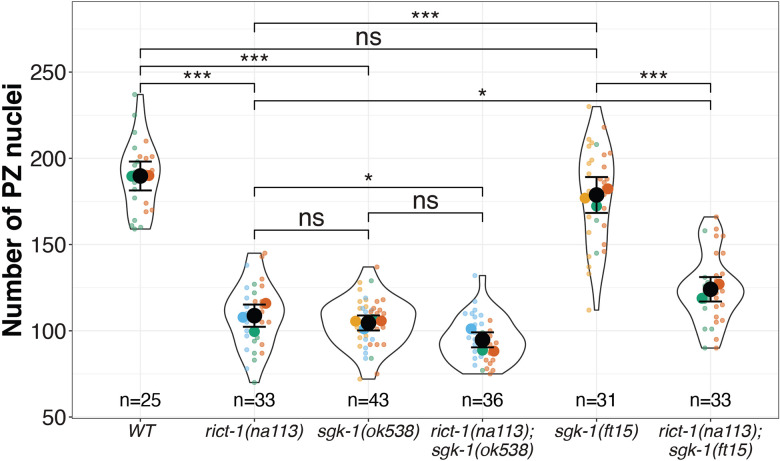
RICT-1 and SGK-1 are likely acting in a linear pathway. Black circles indicate the means across independent replicate experiments (shown in different colors), while the smallest circles indicate individual gonad arms (n = number of gonad arms scored). The violin indicates the overall distribution of data points. Error bars indicate the standard error of the mean. Statistical tests are summarized in Table S2. The asterisks indicate *** p<0.001, ** p<0.01, * p<0.05 of a Linear Mixed Effects Model with a Tukey Post Hoc Test.

In *C. elegans*, SGK-1 acts genetically downstream of Rictor to regulate fat storage, body size, developmental rate (Jones et al., 2009; Soukas et al., 2009), lifespan (Soukas et al., 2009), mitochondrial homeostasis(Gatsi et al., 2014), autophagy (Aspernig et al., 2019), *skn-1* embryonic phenotypes (Ruf et al., 2013), and the transcription of *vit-3* (Dowen et al., 2016). To assayed for non-additive genetic interactions of *rict-1* and *sgk-1* in PZ establishment, we compared PZ counts in the null single and double mutants. We found that the number of PZ nuclei is similar in *rict-1* or *sgk-1* single mutants (108.76 ± 3.17 and 104.54 ± 2.15 for *rict-1(na113)* and *sgk-1(ok538)*, respectively) and in the *rict-1; sgk-1* double mutant (94.74 ± 2.15). These results suggesting that, similar to other *rict-1* phenotypes, *rict-1* and *sgk-1* are likely acting in a linear relationship for this phenotype (Figure 2). While PZ counts in the *rict-1*;*sgk-1* double mutant do not show an additive phenotype in comparison to each single mutant alone, the number of PZ nuclei in the double mutant is significantly lower than in the *rict-1* mutant alone (p<0.05). This result suggests that *sgk-1* may have additional minor effects on this phenotype independent of *rict-1* (Figure 2).

The *sgk-1* gain of function allele (*ft15*) (Jones et al., 2009) suppresses some *rict-1* mutant phenotypes (such as associated learning (Sakai et al., 2017), fat storage, body size, and developmental delay (Jones et al., 2009)). For both autophagy and *skn-1* interactions, *sgk-1(gf)* suppression of *rict-1* mutant phenotypes is partial (Aspernig et al., 2019; Ruf et al., 2013).To determine whether the *sgk-1(ft15) gain-of-function* mutant suppresses *rict-1*, either entirely or partially, we examined *sgk-1(ft15)* alone and in combination with *rict-1(na113).* We found that *sgk-1(ft15)* alone displays a PZ count similar to the wild type (178.74 ± 5.1 versus 189.76 ± 4.06). As expected, if *sgk-1* were acting downstream of *rict-1*, we found a significant, though partial, suppression of the *rict-1* PZ phenotype (to 123.06 ± 3.48 PZ nuclei). Taking the loss- and gain-of-function results together, we conclude that SGK-1 likely acts downstream of RICT-1, even though the two may have other minor independent outputs that affect the PZ phenotype.

### Intestinal RICT-1 contributes to PZ pool establishment

In several different phenotypic contexts, *rict-1* acts from the intestine. These contexts include dauer entry and foraging behavior (O'Donnell et al., 2018), associative learning (Sakai et al., 2017), fat accumulation (Jones et al., 2009; Soukas et al., 2009), and food seeking (McLachlan et al., 2022). Single cell RNA-seq analyses show that *rict-1* mRNA is widely expressed but is most highly abundant in the intestine, (from (Cao et al., 2017; Hutter and Suh, 2016), displayed in Figure S2A). We therefore wondered whether intestinal RICT-1 might influence the germline PZ, even though TORC1 and downstream components did not appear to affect the germ line from the soma (Korta et al., 2012; Roy et al., 2018).

**Figure 3:**
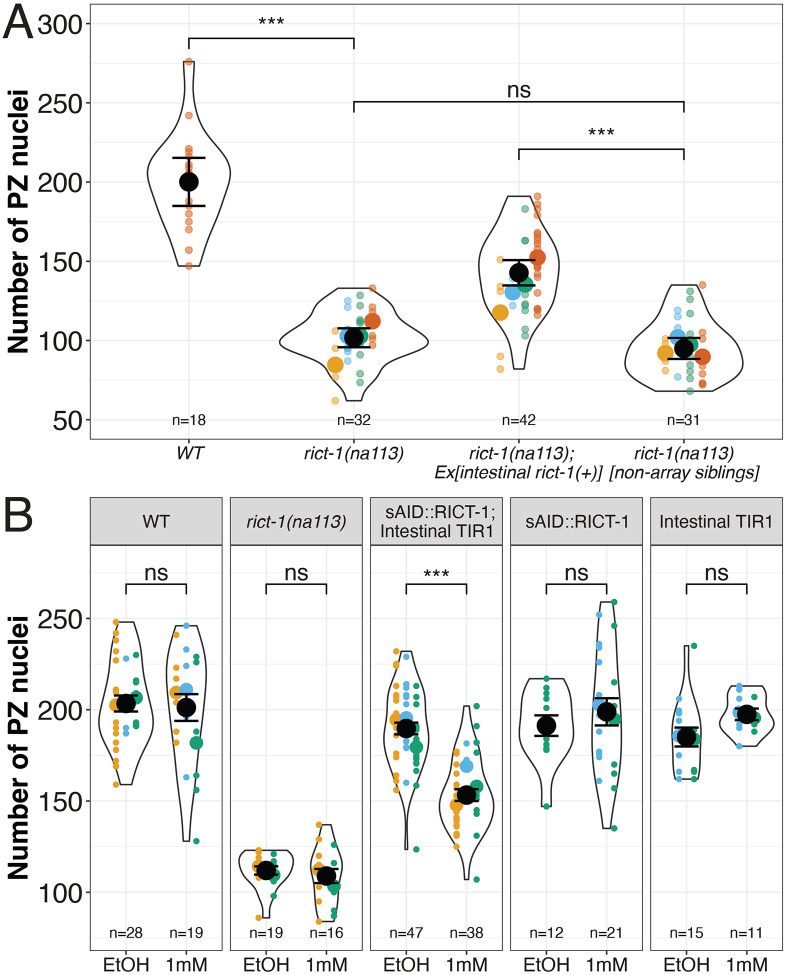
Intestinal RICT-1 is sufficient and necessary for full establishment of the PZ. (A) The intestinal array bearing siblings of *rict-1* (*oyEx713[intestinal rict-1(+)]*) display a significantly higher number of PZ nuclei than their non-array bearing siblings. The non-array bearing siblings are also not significantly different from the non-transgenic *rict-1(na113)* null mutants. (B) In the presence of auxin the Intestinal TIR1 facilitates the degradation of the sAID::RICT-1 (*rict-1*(*na117*)), while the wildtype, the *rict-1(na113)*, the sAID::RICT-1, and the intestinal TIR1 (*ieSi61*) do not display any sensitivity to auxin. Black circles indicate the means across independent replicate experiments (shown in different colors), while the smallest circles indicate individual gonad arms (n = number of gonad arms scored). The violin indicates the overall distribution of data points. Error bars indicate the standard error of the mean. Statistical tests are summarized in Table S2. The asterisks indicate *** p<0.001, ** p<0.01, * p<0.05 of a Linear Mixed Effects Model with a Tukey Post Hoc Test.

To determine whether intestinal *rict-1(+)* is sufficient to promote accumulation of the larval PZ pool, we expressed *rict-1(+)* from an intestinal promoter, *ges-1*, on an extrachromosomal array in the *rict-1(na113)* background. We found that the array-bearing siblings contain a significantly greater number of PZ nuclei (142.74 ± 3.94) than their non-array-bearing siblings (94.97 ± 3.24). In addition, there is no difference between the PZ counts of non-array bearing siblings that have maternal but not zygotic *rict-1* with homozygous *rict-1(na113)* that have neither maternal nor zygotic *rict-1* (Tukey comparison p=0.533). This result supports the conclusion that *rict-1* does not regulate the establishment of the PZ through maternal effects (Figure 3A).

To assess whether PZ pool establishment requires intestinal RICT-1, we depleted RICT-1 in the intestine using tissue-specific auxin-mediated protein degradation (Zhang et al., 2015). We first generated an sAID::RICT-1 fusion (*rict-1(na117)*) by CRISPR-Cas9 genome editing, and determined that it does not impact protein function, as the number of PZ nuclei is not significantly different from that of wild-type worms (Figure S2B). We then crossed it into a strain in which TIR1 is driven by the intestine-specific promoter *ges-1p.* We observed that in the presence of auxin the number of PZ nuclei is significantly reduced (153.26 ± 3.23 with auxin vs. 189.71 ± 3.21 without auxin). Relative to the vehicle (EtOH) treatment, auxin did not affect the PZ counts of the other tested strains tested (wild type, *rict-1(na113)*, *rict-1(na117*[sAID::RICT-1]) or *ieSi61*[*ges-1p*::TIR1] alone), indicating that the lower number of PZ nuclei is due to auxin induced degradation and not auxin induced stress (Figure 3B).

Taken together, our sufficiency and necessity tests suggest that intestinal RICT-1 activity is a major contributor to the accumulation of the larval PZ pool as assayed at the adult molt. Interestingly, intestinally expressed *rict-1* does not entirely rescue to wild-type levels, nor does intestinal degradation of RICT-1 lower the number of PZ nuclei to the level of the *rict-1(na113)* (Figure 3B). These results indicate that RICT-1 acts in additional tissues as well. Single cell RNA-seq analysis (Figure S2A, data from (Cao et al., 2017; Ghaddar et al., 2023; Hutter and Suh, 2016)) indicates that in addition to the intestine, larval *rict-1* is expressed in other tissues, including the germ line. Given our prior results indicating a role for TOR in the germ line (Korta et al., 2012) we tested RICT-1 degradation using *ieSi38*[*sun-1p*::TIR1] and found that it also contributes, though less than the intestine (Figure S2C). We conclude that the intestine is the primary contributing source of RICT-1 important for establishment of the early adult germline PZ, with additional partial contribution from the germ line.

### Neither DAF-7/TGF-ß nor DAF-2/IIS pathways mediate the effects of *rict-1* on the germ line

In the phenotypic context of dauer entry, intestinal *rict-1* signals via neuronal DAF-28 Insulin and DAF-7 TGF-ß from ASI and ASJ (O'Donnell et al., 2018). This finding was of particular interest to us since we previously characterized roles for both the Insulin and TGF-ß pathways in establishing the PZ pool. In both pathways, these same neurons are implicated: *ins-3* is expressed in ASI and ASJ (Ghaddar et al., 2023; Michaelson et al., 2010) and was implicated upstream of DAF-2 for PZ phenotypes, and ASI was implicated by cell ablation for the DAF-7 pathway regulation of the germ line stem cell niche (Dalfo et al., 2012). In both cases, however, the tissue requirements for the receptors and/or downstream effectors were different, and effectors acted independently of neurons and therefore different from their roles in dauer entry (Dalfo et al., 2012; Michaelson et al., 2010; Pekar et al., 2017). Moreover, in our prior studies, the PZ defect seen upon reduced *daf-2* or *daf-7* was fully suppressible by loss of *daf-16* or *daf-3*, respectively.

**Figure 4:**
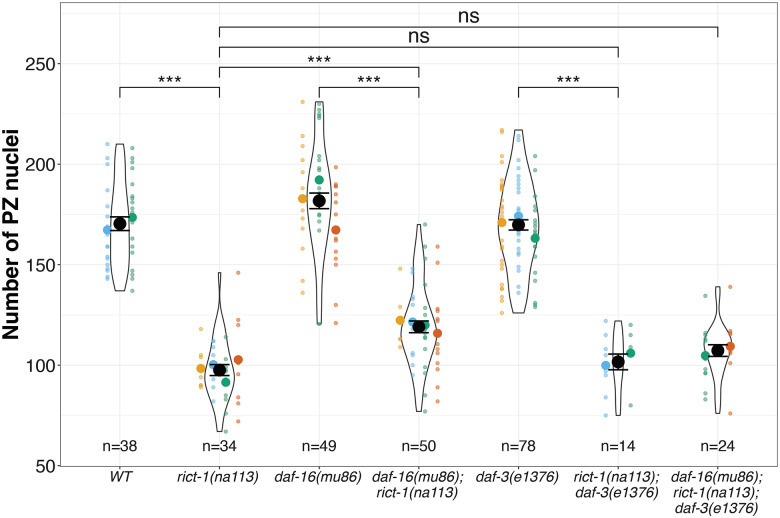
Neither the Insulin pathway nor the DAF-7/TGF-ß pathway mediate the effects of RICT-1 on the germ line. Black circles indicate the means across different replicate experiments (shown in different colors), while the smallest circles indicate individual worms (also the number at the bottom of each graph). The violin displays the overall distribution of data points. Error bars indicate the standard error of the mean. Statistical test results are summarized in Table S2. The asterisks indicate *** p<0.001, ** p<0.01, * p<0.05 of a Generalized Linear Model with a Tukey Post Hoc Test.

If the relevant IIS and/or TGFß pathways mediate the effects of *rict-1*, we would expect *daf-16* and/or *daf-3* to suppress the *rict-1* mutant PZ defect. To test this hypothesis, we built double and triple mutants of *rict-1(na113)* with *daf-16(mu86)* and *daf-3(e1376)* and counted the PZ pool. We found that neither *daf-16(mu86)* nor *daf-3(e1376)* alone strongly suppressed the PZ defect in the *rict-1* mutant. The PZ of the *daf-16(mu86); rict-1(na113)* averaged 119.09 ± 2.91 (compared with 97.57 ± 2.73 for *rict-1(na113)* alone. While this small difference is statistically significant, we note that *daf-16(mu86)* has a greater (though not statistically significant) average number of PZ nuclei than the wild type (Figure 4). Most important, the triple mutant *daf-16(mu86); rict-1(na113); daf-3(e1376)* averages the same number of PZ nuclei (107.27 ± 2.89) as either double mutant or the *rict-1(na113)* mutant alone (Figure 4). We conclude from these results that neither *daf-16* nor *daf-3* are negatively regulated downstream of RICT-1 as it impacts PZ accumulation.

### Transcripts of four vitellogenin genes are depleted in *rict-1* relative to wild type at the mid-L4

**Figure 5:**
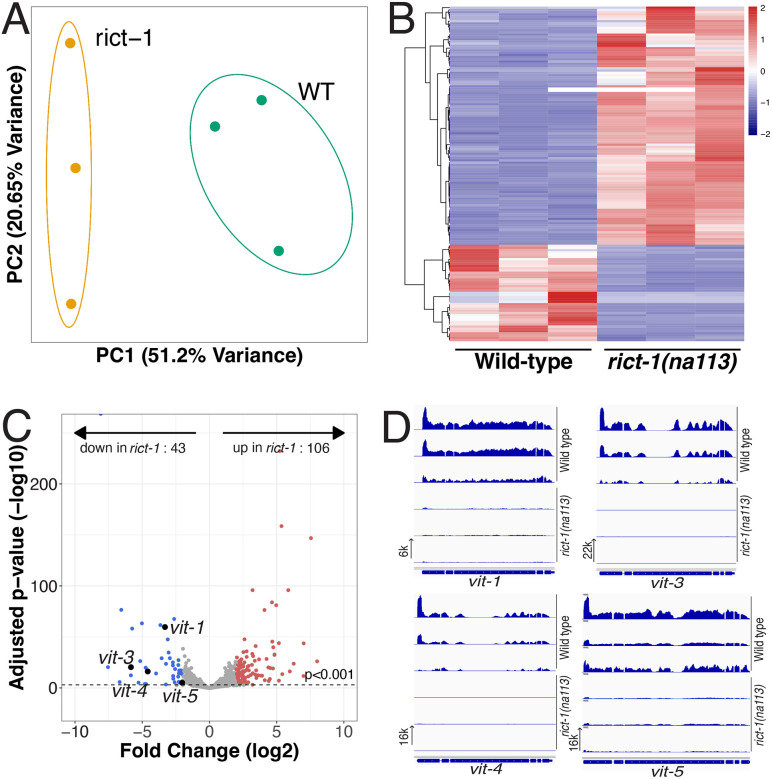
Significant differences in *rict-1* mutant versus wild-type transcript abundance in L4 larvae include four vitellogenin genes. (A) Principal Component Analysis of the two strains, in which each circle indicates one library, color coded by the two different strains. (B) A heatmap in which each line indicates one differentially regulated gene (log2 fold change >2 and adjusted p value < 0.001) between the *rict-1(na113)* mutant and the wild type. Darker colors indicate a stronger differential, where blue colors indicate lower and red higher abundance. (C) A volcano plot displaying genes in (B) as circles in both the *rict-1(na113)* mutant and the wild-type: not significantly regulated (grey), down-regulated in the *rict-1(na113)* mutant (red) or up-regulated in the *rict-1(na113)* mutant (blue) relative to the wild type. Vitellogenin genes are labeled and marked in larger black circles. The dashed line indicates the adjusted p-value threshold of <0.001. (D) The transcription pattern of the 4 differentially regulated vitellogenin genes is displayed in the Integrative Genome Viewer (Robinson et al., 2011). In each case, the top 3 rows are the wild type, and the lower 3 rows are the *rict-1(na113)* mutant; the transcript abundance scale is shown for each gene on the left side of the bottom row.

To determine how *rict-1* may be influencing the larval germ line, we performed comparative bulk RNA-sequencing of tightly synchronized mid-L4 stage *rict-1* mutants and wild-type worms in biological triplicate. We detected 14,153 protein coding transcripts (see Methods). Out of these, 154 are differentially regulated (adjusted p < 0.05 and log2 fold change >2) between the wild type and the *rict-1(na113)* mutant (110 less and 44 more abundant in the *rict-1(na113)* mutant than in the wild type). Applying a more stringent adjusted p value cut off of p<0.001 (log2 fold change >2) we detect 149 differentially regulated transcripts, out of which 106 are more and 43 are less abundant in the *rict-1(na113)* mutant than in the wild type. (Figure 5). Our results indicate that genotype accounts for strongest variance, and that the replicates identify similar sets of differentially abundant transcripts (Figure 5A, B). Complete transcript counts and statistics are in Table S3.

Of particular interest were genes expressed in the intestine, and, among these, vitellogenin genes caught our attention for several reasons. The *C. elegans* genome encodes six vitellogenins, members of a family of conserved yolk proteins (Perez and Lehner, 2019) best described for their adult function in provisioning oocytes (Grant and Hirsh, 1999; Kimble and Sharrock, 1983). However, no function in larval stages or the larval germ line is yet described. We found that four of the six vitellogenins (*vit-1, −3, −4*, and −*5*) are significantly lower in mid-L4 larval *rict-1* mutants relative to wild type (p<0.001, log2 fold change < −2) (Figure 5C,D)

### VIT-3 promotes germline progenitor pool establishment

**Figure 6:**
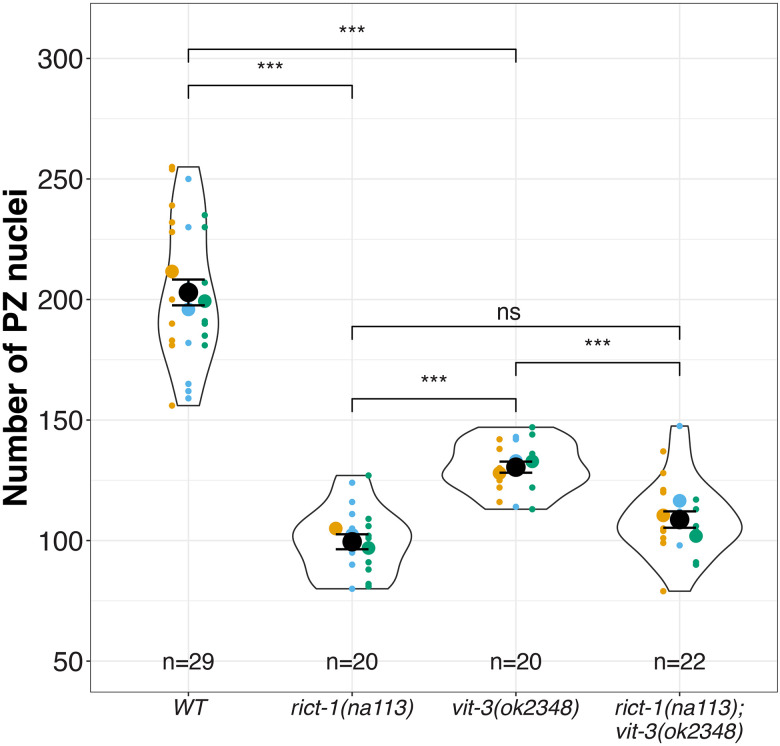
*rict-1* and *vit-3* are likely acting in a linear pathway. Black circles indicate the means across different replicate experiments (shown in different colors), while the smallest circles indicate individual gonad arms (n = number of gonad arms scored). The violin displays the overall distribution of data points. Error bars indicate the standard error of the mean. Statistical tests are summarized in Table S2. The asterisks indicate *** p<0.001, ** p<0.01, * p<0.05 of a Generalized Linear Model with a Tukey Post Hoc Test.

Among the vitellogenin genes we detected in our comparative RNA-seq analysis, *rict-1* was previously found to indirectly regulate *vit-3* transcription in the adult intestine (Dowen, 2019; Dowen et al., 2016). We therefore tested whether *vit-3* could, like *rict-1*, contribute to accumulation of the larval PZ pool. To this end, we counted the number of PZ nuclei in worms bearing a loss-of-function *vit-3* mutation. If VIT-3 were important for PZ establishment we would expect that the PZ pool would be depleted relative to wild type. We observed that the *vit-3(ok2348)* null mutant contains significantly fewer PZ nuclei (130.45 ± 2.3) than the wild type (202.93 ± 5.35) (Figure 6). If *rict-1* and *vit-3* were acting in a linear genetic pathway, we would expect that loss of both *rict-1* and *vit-3* would not further deplete the PZ pool beyond either single mutant alone. We found that the double *rict-1; vit-3* mutant has the same number of PZ nuclei as the *rict-1(na113)* mutant alone (108.7 ± 3.4 vs 99.5± 3, respectively). Together, these results reveal an unexpected role for *vit-3* in the larval accumulation of germline progenitors, likely downstream of *rict-1*.

## DISCUSSION

We generated seven new alleles of *rict-1*, including six similarly behaving alleles encoding small deletions or early stop codons, and one encoding a fully functional AID-tagged RICT-1 protein. Using these and prior existing SNP alleles, we show that *rict-1* is required for several aspects of reproduction, including timing of progeny production. We focused on a larval requirement for *rict-1* in germline development: the accumulation of the germline progenitor cells that establishes the early adult steady-state germline progenitor pool. We determined that the *rict-1* mutant PZ pool reaches only half the normal number of cells at the adult molt. We observed a similar phenotype upon loss of *sgk-1*, a canonical effector downstream of *rict-1*, with no additional defect in the double mutant and partial but significant suppression of *rict-1* by an *sgk-1(gf)* allele. We also discovered a prominent role for intestinal *rict-1* in larval establishment of the PZ that is both sufficient and necessary for establishment of the full complement of wild-type PZ cells. Despite other phenotypic contexts where *rict-1* depends on pathways previously implicated in PZ pool regulation, we find no obvious genetic interaction with the insulin or DAF-7/TGFß pathways. Finally, we identify four vitellogenin genes among transcripts that are highly reduced in *rict-1* L4 larvae relative to wild type, and demonstrate a role for one of them, *vit-3*, in promoting the progenitor pool, likely downstream of *rict-1*.

Using multiple mutant alleles of *rict-1*, we confirmed low broods reported by others (Jones et al., 2009; Soukas et al., 2009), and we also observe an extended timeline of progeny production and no increase of progeny production upon replete sperm provision. In mated worms, brood size correlates with the number of cells in the progenitor zone (PZ) (Agarwal et al., 2018; Kocsisova et al., 2019). Multiple *rict-1* mutants affecting different areas of the protein all displayed similarly reduced numbers of PZ cells (~half of the wild type number). These alleles alter coding sequences near the N-terminus (*na116* and *na119*), the third exon (*ft7*, *na112*, and *na113*) and the 11^th^ exon (*mg360*, *na114*, and *na115*). The N-terminus and third exon mutants disrupt isoform A and B (Figure 1C), but nevertheless display the same PZ phenotype as those mutants that affect all three isoforms. This suggests that the C isoform is unlikely to play a role in the establishment of the PZ. We also found that the mitotic index of PZ cells in *rict-1* mutants did not differ from the wild type (Figure 1D), indicating that *rict-1* is unlikely affecting the establishment of the PZ by markedly slowing cell cycle progression. Exactly how loss of *rict-1* affects the PZ pool at the cellular level, and whether it affects germline stem cell fate is of future interest.

Our results suggest that, as in other phenotypic contexts, RICT-1 acts upstream of SGK-1 to regulate the PZ. Similar to reports for *rict-1* phenotypes affecting fat storage, body size, developmental rate (Jones et al., 2009; Soukas et al., 2009), lifespan (Soukas et al., 2009), mitochondrial homeostasis (Gatsi et al., 2014), autophagy (Aspernig et al., 2019), and *skn-1* in embryonic development (Ruf et al., 2013), we see no additive effect of a *rict-1; sgk-1* double mutant over either single mutant. We also tested a *sgk-1 (gf)* allele (Jones et al., 2009), that suppresses some *rict-1* phenotypes entirely (such as those associated with learning (Sakai et al., 2017), fat storage, body size, and developmental delay (Jones et al., 2009). Significant, though partial, suppression of *rict-1* mutant phenotypes were also reported for with phenotypes associated with autophagy (Aspernig et al., 2019) and *skn-1* interactions (Ruf et al., 2013). As in the latter cases, we observed significant, though partial, suppression of the *rict-1* mutant PZ defect. These results suggest that *rict-1* may have additional downstream outputs onto relevant effectors for its role in establishing the PZ pool.

Our results show that intestinal RICT-1 is both sufficient and necessary for full establishment of the PZ pool. RICT-1 intestinal sufficiency has been demonstrated for other phenotypes including entry to the dauer stage, foraging behavior (O'Donnell et al., 2018), associative learning (Sakai et al., 2017), fat accumulation (Jones et al., 2009; Soukas et al., 2009), and food seeking (McLachlan et al., 2022)). Tagging RICT-1 at its endogenous locus with AID, we used the auxin degradation system to also assay the requirement for intestinally produced *rict-1* in an otherwise wild-type background (Figure 3B). Because auxin exposure prolongs lifespan (Loose and Ghazi, 2021) and promotes resistance to endoplasmic reticulum stress (Bhoi et al., 2021), we used a minimal concentration of auxin (1mM) that did not enhance the PZ defect in the *rict-1* mutant nor in other control strains. Our results indicate that the intestinal rescue of *rict-1(+)* in the *rict-1(na113)* background does not rescue the number of PZ nuclei to the wild-type level, and that the intestinal degradation does also not reduce the number PZ nuclei from the wild-type level to the *rict-1(na113)* level. Results from scRNA-seq of larvae (Cao et al., 2017; Hutter and Suh, 2016) indicate that *rict-1* expression is broad but is highest in the intestine, followed by the germ line (Figure S2A), where we also see a partial requirement (Figure S2C). Whether the intestine and germ line are the only required tissues, and how the germline-autonomous role interacts with TORC1 function are areas for further investigation.

In the phenotypic context of dauer entry, RICT-1 acts via both the DAF-28/Insulin and the DAF-7/TGF-ß in the ASI and ASJ neurons (O'Donnell et al., 2018). However, we observed that neither the insulin nor TGFß signaling pathways, both of which are implicated in larval PZ pool expansion (Dalfo et al., 2012; Michaelson et al., 2010; Pekar et al., 2017), act downstream of RICT-1 to promote PZ pool expansion.

The results of our RNA-seq and genetic analysis suggest that *rict-1* in L4 larvae transcriptionally regulates at least four of the six vitellogenin genes, and that a *vit-3* mutant has a PZ defect similar to but not as severe as loss of *rict-1*. No function has been previously ascribed for vitellogenins in post embryonic development nor in the germ line prior to adult oocyte provision (reviewed in (Perez and Lehner, 2019)). RICT-1 indirectly promotes high levels of *vit-3* transcription in the adult intestine (Dowen et al., 2016). In this scenario, adult transcriptional activation of *vit-3* occurs in response to heterochronic cues and is controlled by CEH-60, a protein that can also act as a transcriptional repressor of stress response genes, suggesting a tradeoff between larval stress response and adult reproduction (Dowen, 2019; Dowen et al., 2016).

Nevertheless, our results point to a possibly different role for vitellogenins, prior to oocyte provisioning. In combination with the high fat phenotype of the *rict-1* mutant (Jones et al., 2009; Soukas et al., 2009; Yen et al., 2010) we speculate that RICT-1 regulates the establishment of the PZ via intestine-germline transport of lipids or other molecules associated with vitellogenin-containing lipid particles. Vitellogenins are secreted from the adult intestine into the body cavity (Kimble and Sharrock, 1983), pass through gonad sheath pores (Hall et al., 1999), and are taken up by developing oocytes via receptor-mediated endocytosis (Grant and Hirsh, 1999). Molecules associated with vitellogenins could act as a nutrient source for post-embryonic survival (Perez and Lehner, 2019). The expression of all six vitellogenins is negligible during the first three larval stages in comparison to the rapidly increasing expression during the fourth larval stage and adulthood (Cao et al., 2017; Ghaddar et al., 2023; Hutter and Suh, 2016), a time-frame that correlates with the rapid expansion of the PZ pool (Killian and Hubbard, 2005). We note that although loss of *vit-3* causes a mutant phenotype is somewhat less severe than *rict-1*, this result suggests that other *vits* from our RNA-seq analysis may contribute to PZ expansion.

What might vitellogenins provide to the larval germ line? One possibility is that they facilitate transport of a critical nutrient lipids, such as cholesterol. Another possibility is that they provide limiting membrane lipids required for the demands of germ cell division. Yet another possibility is that they associate with as-yet unknown moieties such as hormones to direct germline development. The oocyte receptor for vitellogenins, RME-2, is not detectable in the distal gonad during larval stages (Grant and Hirsh, 1999; Salinas et al., 2007). However, a function for RME-2 as a high affinity receptor prior to oogenesis has not been formally ruled out. Alternatively, a different receptor may fulfill this role in larvae. Additional studies are required to determine how RICT-1 regulates larval vitellogenins and how vitellogenins participate in the establishment of the PZ pool.

In conclusion, we present the first evidence that the TORC2 component RICT-1 contributes to establishment of the full germline stem/progenitor pool. Our results suggest a model whereby intestinal RICT-1 promotes the expression of vitellogenins that support germline expansion during larval stages. Due to the high conservation of TORC2 components, vitellogenins, and their receptors, our results may have general implications for how Rictor and TORC2 regulate germline development in other organisms and, more generally, how they may support pools of proliferating stem or progenitor cells in other organisms and organ contexts. Moreover, since vitellogenin-like molecules, such as ApoB, are implicated in heart disease and other aspects of lipid metabolism, the *C. elegans* larval germ line may provide in roads to the identification of additional associated moieties and receptors.

## MATERIALS AND METHODS

### Worm maintenance and husbandry

*C. elegans* nematodes were grown on NGM solid media at 20°C and fed *Escherichia coli* strain OP50 (Stiernagle, 2006). All strains generated and used in this study are listed with full genotypes in Table S1. Some strains were provided by the CGC. All strains were based on Bristol N2 (RRID:WBSTRAIN:WBStrain00000001). Many experiments required access to information on WormBase (Sternberg et al., 2024).

### Determination of progenitor zone nuclei counts and mitotic index

Mid-L4 worms (L4.5; (Mok et al., 2015)) were picked from mixed stage plates and transferred to new plates. 6h later all early adult worms bearing a vulval slit were picked into 1ml of M9+Tween within a 1.5ml of low-retention microfuge tube (02-681-320, Fisherbrand). Worms were then spun down on a table-top centrifuge for 10 seconds, before the supernatant was carefully removed with a pipette under the dissecting scope, to avoid loss of worms. Another 500μl of M9+Tween was added, followed by a centrifugation and supernatant removal. 50μl of 100%EtOH were added to each sample, and worms were allowed to settle at the bottom for ~10min. After 10min as much EtOH was removed as possible without disturbing the worms. Finally, ~5μl of Vectashield+DAPI (H-1000, Vector Laboratories) was added, and worms were gently pipetted onto a 4% agar pad on a glass slide. Slides were kept in the cold and dark and were imaged within 2 weeks after staining. Imaging was performed on Nikon W1 spinning disk confocal microscope with 60x oil immersion magnification with z-stacks of 0.5μm step size. Only one gonad arm was counted per individual worm. The collected files were counted with ImageJ(Arganda-Carreras et al. 2024) to manually count the number of PZ nuclei. Mitotic index presented as a percentage of the number of mitotic figures (identified as characteristic metaphase and anaphase figures) divided by the total number of PZ nuclei.

### Brood Size Determination

L4 worms were picked from mixed stage plates. Single hermaphrodites were added to plates and, for the mated treatment, were joined by 3 wild-type males, which were replaced every two days. All worms were transferred every day to new plates, and hatched offspring were counted 2 days later.

### CRISPR-Cas9 genome editing

CRISPR/Cas9 genome editing was performed using pre-incubated Cas9 (Berkeley)::(crRNA +tracrRNA) (IDT) ribonucleoprotein, and screening was facilitated by co-CRISPR detection using the *dpy-10(cn64)* mutation as described (Paix et al., 2016). DNA repair templates contained ~25–35 bps of homology on each arm as ssDNA oligos (<150 bps) (IDT). The guide RNAs and oligos are listed in Table S4. For Stop-In mutants (*na112, na113, na114*, and *na115*) the inserted stop cassette led to an early stop in all frames (Wang et al., 2018) .

### RNA extraction for bulk RNA-seq

Worms were bleached and synchronized as previously described (Stiernagle, 2006). Synchronized L1 larvae were added to 15 10cm NGM plates (each inoculated with 1ml of OP50) at different times, so that all strains would reach the mid-L4 stage at the same time (as previously empirically determined). Worms were washed off the plates with RNAse-free water (10977015, Invitrogen) and collected atop pluristrainers with 40μl mesh size (#43-10040-40 from Pluristrainer.com) and washed three times with 5ml of RNAse free H_2_0. Pluristrainers were turned over, and 1ml of RNAse-free water was used to wash the worms off the mesh and collect them in RNAse-free 2ml Eppendorf microfuge tubes (E0030123620, Eppendorf). Each 1ml of packed worms was resuspended in 4ml of Trizol. The worm+Trizol mix was vortexed and then carefully dropped into liquid nitrogen and then thawed at 37°C. After three freeze-thaw cycles, samples were thawed at 37°C, and 2ml of Trizol+2ml of Chloroform/packed ml of worms was added and worms and shaken by hand. Samples were aliquoted into 2ml RNAse-free tubes and incubated at room temperature for three minutes. Samples were then spun at 12,000g at 4°C for 15 min. The aqueous layer was removed into new RNAse-free tubes, and an equal volume of 70% ethanol was added and inverted to mix. This solution was transferred to a spin column (RNeasy Qiagen kit # 74104) and was spun down at 21k rpf for 30sec. The column was washed once with buffer RW1 and centrifuged for 15 seconds at 21k rpf DNase I was added directly to each RNeasy column membrane and was left to incubate at room temperature for 15 minutes. Each column was washed with Qiagen RW1 and RPE buffers, before the sample was eluted from the spin column in RNase-free water to be collected in a fresh 2ml RNase-free tube. RNA quality was initially assessed with a nanodrop (NanoDrop 8000 UV-Vis Spectrophotometer). When samples were submitted to the NYU genome technology core. The automated RNA-seq library preparation and polyA selection was carried out before samples were analyzed with the NovaSeq X.

### RNA-seq analysis

Version WBCel235 of the *C. elegans* genome was used to map sequencing reads. Seq-N-Slide was used to align the sequencing results and determine those gene transcripts that are differentially detected between the mutant and wild-type strains (Dolgalev). Transcript counts were restricted to protein coding genes (19,983 genes). Prior to further analysis this count data was further restricted to include only genes with counts-per-million >1 in at least two out of the six libraries (14,153 genes). Principal component analysis (PCA) was performed on log2 normalized CPM values on all genes included in the differential gene expression analysis (14,153 genes), using the stats package in R (version 2024.04.2+764) (RStudioTeam, 2020). Lists of genes with differentially abundant transcripts are in Table S3, along with summary statistics. The entire dataset will be publicly available upon publication at NCBI Gene Expression Omnibus.

### Statistical analyses

Plots in Figures 1-6 and Figures S1-S2 were generated using R with minor modifications using Adobe Illustrator. Statistical analysis was conducted in R (version 2024.04.2+764) (RStudioTeam, 2020). All PZ counts and the offspring data were analyzed with a Generalized Mixed Effect Model (Nelder and Wedderburn, 1972), taking the replicate as a random factor into account. The Mitotic Index data was analyzed using a Linear Model (Nelder and Wedderburn, 1972), and the Reproductive Period data was analyzed with a Linear Mixed Effects Model (Nelder and Wedderburn, 1972).

## Supplementary Material

Supplement 1
