# Supplementary material for: Intestinal RICT-1 regulates the larval germline progenitor pool via the vitellogenin VIT-3 in C. elegans
Source: bioRxiv. 2025 Jan 9:2025.01.08.632040. Preprint. [Version 1] doi: 10.1101/2025.01.08.632040 (PMC11741266; doi:10.1101/2025.01.08.632040)
Supplement: Supplement 1 [file media-1.pdf]

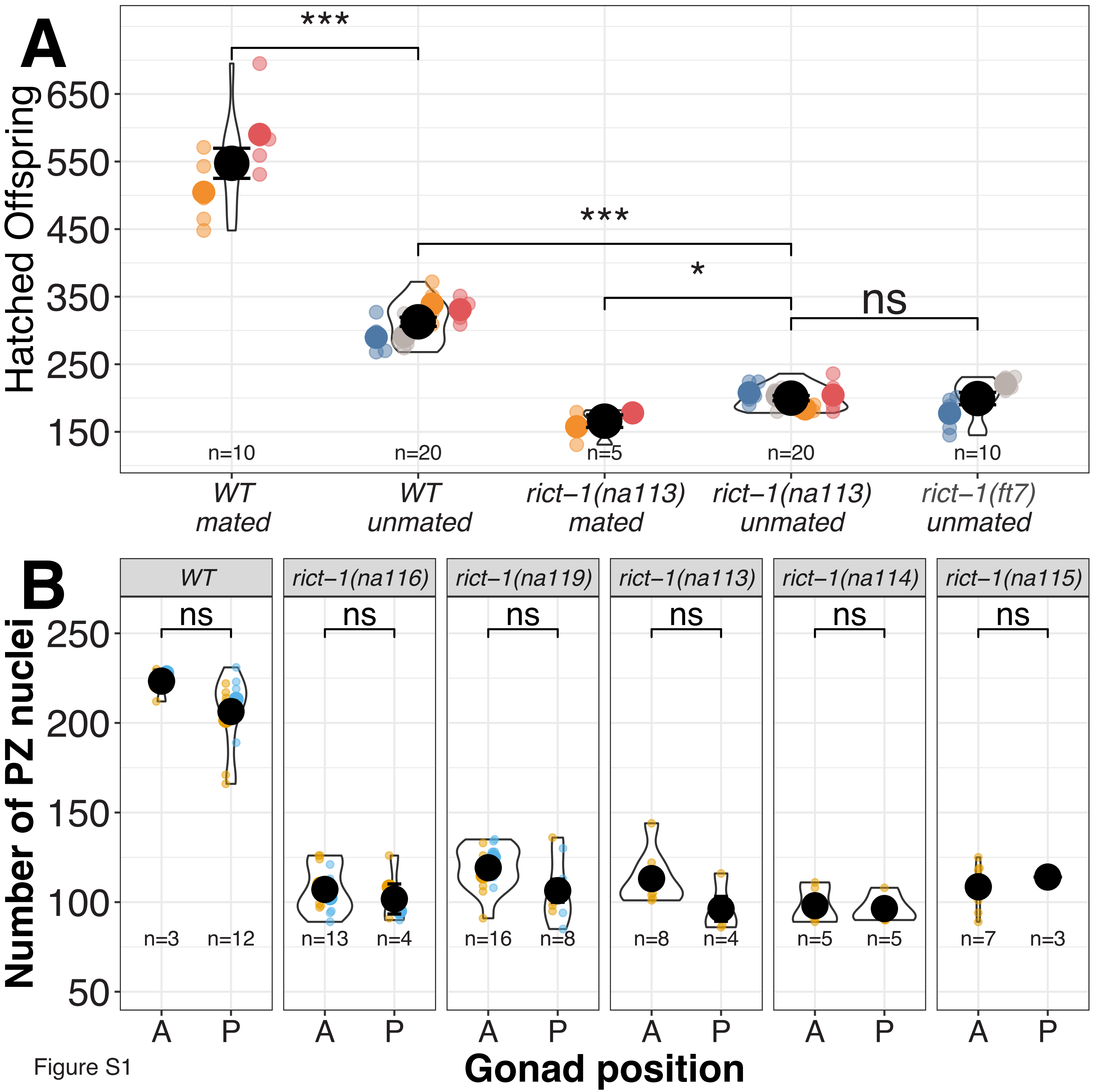
A
Hatched Offspring
650
550
450
350
250
150
***
***
*
ns
n=10
n=20
n=5
n=20
n=10
WT mated
WT unmated
rict−1(na113) mated
rict−1(na113) unmated
rict−1(ft7) unmated
B
Number of PZ nuclei
WT
rict−1(na116)
rict−1(na119)
rict−1(na113)
rict−1(na114)
rict−1(na115)
ns
250
200
150
100
50
n=3
n=12
n=13
n=4
n=16
n=8
n=8
n=4
n=5
n=5
n=7
n=3
A
P
Gonad position

Figure S1

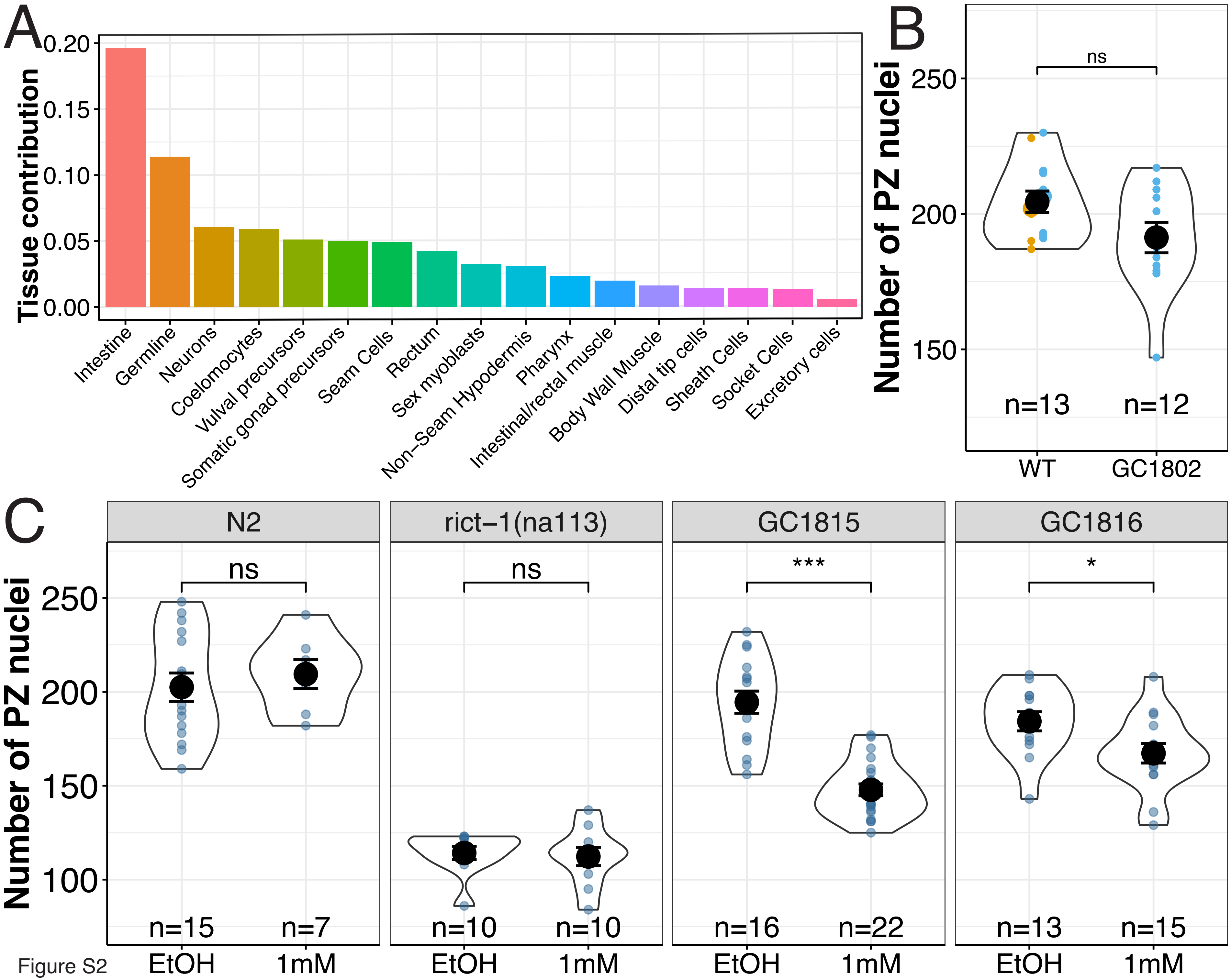
A
B
C
Tissue contribution
0.20
0.15
0.10
0.05
0.00
Intestine
Germline
Neurons
Coelomocytes
Vulval precursors
Somatic gonad precursors
Seam Cells
Rectum
Sex myoblasts
Non−Seam Hypodermis
Pharynx
Intestinal/rectal muscle
Body Wall Muscle
Distal tip cells
Sheath Cells
Socket Cells
Excretory cells
Number of PZ nuclei
250
200
150
ns
n=13
n=12
WT
GC1802
N2
rict−1(na113)
GC1815
GC1816
ns
ns
***
*
100
n=15
n=7
n=10
n=10
n=16
n=22
n=13
n=15
EtOH
1mM
EtOH
1mM
EtOH
1mM
EtOH
1mM
Figure S2

SUPPLEMENTAL FIGURE LEGENDS

Figure S1: Mated *rict-1* mutants do not produce more offspring, and gonad position does not affect PZ counts. (A)Number of hatched offspring from unmated hermaphrodites and mated hermaphrodites. Mated wild-type worms produce more offspring than unmated wildtype worms, while the *rict-1(na113)* mutant has fewer offspring when mated, partially due to poor survival. The data for unmated worms here are the same as those displayed in Figure 1F. Worms were mated with N2 (in orange) or CB4855 (in red) males, but the male strain conferred no difference. Mating was confirmed by abundant male offspring and/or mating plug. (B) The number of PZ nuclei does not depend on the gonadal position in neither the wild-type nor five different *rict-1* mutants. For a subset of the samples in Figure 1B gonadal position was noted, where A indicates anterior gonad arm and P indicates posterior gonad arm. (A, B) Black circles indicate the means across different replicate experiments (shown in different colors), while the smallest circles indicate individual gonad arms (n = number of gonad arms scored). The violin indicates the overall distribution of data points. Error bars indicate the standard error of the mean. Two-sided t-tests were performed, and the significances are noted as follows: *** $p<0.001$, ** $p<0.01$, * $p<0.05$.

Figure S2: RICT-1 is expressed in different tissues with different contributions, the *na117* [sAID::RICT-1] fusion does not affect the PZ phenotype, and RICT-1 is acting from the germline albeit at a smaller effect than from the intestine. (A) Proportion of *rict-1* transcripts as a function of tissue assignment from L2 scRNA-seq (Cao et al., 2017; Hutter and Suh, 2016). The relative contribution of each tissue is shown. (B) Number of PZ nuclei in wild type and GC1802 *rict-1(na117*[sAID::RICT-1]); data here are the same as in Figure 3B for the two strains on EtOH. (C) Number of PZ nuclei in wild type and strains that deplete RICT-1: GC1815 carries *rict-1(na117* [sAID::RICT-1]) and *ieSi61* [ges-1p::TIR1] while GC1816 carries *rict-1(na117* [sAID::RICT-1]) and *ieSi38* [sun-1p::TIR1]. (B,C) Black circles indicate the means across different replicate experiments (shown in different colors), while the smallest circles indicate individual gonad arms (n = number of gonad arms scored). The violin indicates the overall distribution of data points. Error bars indicate the standard error of the mean. The asterisks indicate *** $p<0.001$, ** $p<0.01$, * $p<0.05$ of two-sided Student's T Test.

SUPPLEMENTAL TABLES

Table S1: Worm strains generated and used in this study

Strain	Genotype	Primers used (Details in Table S4)	Reference/Source and/or parent strain
BR2742	*pept-1(lg601) X*	GCo2856 + GCo2857	CGC (Meissner et al., 2004)
CA1209	*ieSi61 II; unc-119(ed3) III*	GCo3081 + GCo3202	CGC (Zhang et al., 2015)
CB1376	*daf-3(e1376) X*	GCo2044 + GCo2045	CGC (Riddle et al., 1981)
CB4855	*Wild type – cross*	NA	CGC (Hodgkin and Doniach, 1997)
CF1038	*daf-16(mu86) I*	GCo3147 + GCo3148 + GCo3149	CGC (Lin et al., 1997)
GC1745	*rict-1(na112)* II	GCo2979 + GCo2980	This work, CRISPR
GC1746	*rict-1(na113)* II	GCo2979 + GCo2980	This work, CRISPR

GC1747	*rict-1(na114)* II	GCo2993 + GCo2994	This work, CRISPR
GC1756	*rict-1(na113)* II*; oyEx713[ges-1p::rict-1cDNA]*	GCo2979 + GCo2980	GC1746, PY9313
GC1759	*daf-16(mu86) I; rict-1(na113) II*	GCo2979 + GCo2980<br>GCo3147 + GCo3148 + GCo3149	GC1746, CF1038
GC1762	*rict-1(na113) II; daf-3(e1376) X*	GCo2979 + GCo2980<br>GCo2044 + GCo2045	GC1746, CB1376
GC1764	*rict-1(na115)* II	GCo2993 + GCo2994	This work, CRISPR
GC1779	*rict-1(na113) II; sgk-1(ft15) X*	GCo2979 + GCo2980<br>GCo3070 + GCo3071	GC1746, KQ1564
GC1780	*rict-1(na113) II; sgk-1(ok538) X*	GCo2979 + GCo2980<br>GCo3072 + GCo3073	GC1746, VC345
GC1793	*daf-16(mu86) I; rict-1(na113) II; daf-3(e1376) X*	GCo2979 + GCo2980<br>GCo3147 + GCo3148 + GCo3149<br>GCo2044 + GCo2045	GC1762, CF1038
GC1800	*rict-1(na113) II; pept-1(lg601) X*	GCo2979 + GCo2980<br>GCo2856 + GCo2857	GC1746, BR2742
GC1801	*rict-1(na116) II*	GCo3040 + GCo3018	This work, CRISPR
GC1802	*rict-1(na117) II*	GCo3040 + GCo3018	This work, CRISPR
GC1815	*ieSi61 rict-1(na117) II; unc-119(ed3) III*	GCo3040 + GCo3018<br>GCo3081 + GCo3202	GC1802, CA1209
GC1816	*rict-1(na117); unc-119(ed3) III; ieSi38 IV*	GCo3040 + GCo3018<br>GCo3202+GCo3275	GC1802, CA1199
GC1830	*rict-1(na119) II*	GCo3040 + GCo2980	This work, CRISPR
GC1842	*rict-1(na113) II; vit-3(ok2348) X*	GCo2979 + GCo2980<br>GCo3223 + GCo3224	GC1746, RB1815
KQ6	*rict-1(mg360)* II	GCo2993 + GCo2994	CGC(Jones et al., 2009)
KQ1366	*rict-1(ft7)* II	GCo2979 + GCo2980	CGC (Jones et al., 2009)
KQ1564	*sgk-1(ft15) X*	GCo3070 + GCo3071	CGC (Jones et al., 2009)
N2	Wild Type	NA	CGC
PY9313	*rict-1(ft7)* II*; oyEx713[ges-1p::rict-1cDNA]*	GCo2979 + GCo2980	(O'Donnell et al., 2018)
RB1815	*vit-3(ok2348) X*	GCo3223 + GCo3224	CGC (The C. elegans Deletion Mutant Consortium 2012)
VC345	*sgk-1(ok538) X*	GCo3072 + GCo3073	CGC (The C. elegans Deletion Mutant Consortium 2012)

Table S2 Summary results from statistical tests

Figure	Statistical Model	Variable	Factor	Random Factor	Additional Info	Chi sq	Degrees of Freedom	Pr(>Chisq)	Sig
1B	LMEM	Number of PZ nuclei	Strain	Replicate	-	968.51	8	< 2.2e-16	***
1C	LM	Mitotic Index	Strain	-	Sum Sq= 9.005, F value = 1.0382	-	5	0.401	ns
1F	LMEM	Sum of hatched Offspring	Strain	Replicate	-	246.59	2	< 2.2e-16	***
1G	GLM	Hatched offspring over Time	Strain	-	family = poisson	646.69	2	< 2.2e-16	***
1H	LMEM	Reproductive Period	Strain	Replicate	-	34.916	2	2.62E-08	***
2	LMEM	Number of PZ nuclei	Strain	Replicate	-	666.43	5	< 2.2e-16	***
3A	LMEM	Number of PZ nuclei	Strain	Replicate	-	227.72	3	< 2.2e-16	***
3B	LMEM	Number of PZ nuclei	Strain and [Auxin]	Replicate	-	58.74	9	< 2.2e-16	***
4	LMEM	Number of PZ nuclei	Strain	Replicate	-	679.9	6	< 2.2e-16	***
6	LMEM	Number of PZ nuclei	Strain	Replicate	-	434.05	3	< 2.2e-16	***

LMEM = Linear Mixed Effects Model

LM = Linear Model
GLM = Generalized Linear Model

Table S3:
RNAseq

Table S4: Oligos used for CRISPR injections

Plasmid/Oligo	Strain	Method used, result	Injected into	Guide used	Co-CRISPR
GCo3335	*GC1745 (rict-1(na112))* *GC1746 (rict-1(na113))*	CRISPR, early stop in	N2	GCo3334	dpy-10
GCo3337	*GC1747 (rict-1(na114))* *GC1764 (rict-1(na115))*	CRISPR, early stop in	N2	GCo3336	dpy-10
NA	GC1801 (*rict-1(na116)*)	CRISPR, deletion	N2	GCo3348	dpy-10
Gco3069	GC1802 (*rict-1(na117)*)	CRISPR, sAID::RICT-1	N2	GCo3348	dpy-10
NA	GC1832 (*rict-1(na119)*)	CRISPR, deletion	N2	GCo3349	dpy-10

Table S4: List of Oligos

Primers	Sequence
Gco2044	gccgtttctggtttgacacctgg
Gco2045	cggcaggatccagttgacgatatg
Gco2856	acaacccgcatcaaaacttggtctccaacag
Gco2857	cgctgggaattagattaatcaacggctgacttag
Gco2979	tgttaaaattcctacccgacgatcacccgt
Gco2980	acttttcagatttattctgcaacgaccaga
Gco2993	atacttctctgggagttcaggcgccattgc
Gco2994	tcgcatatcggaacctattggattcccatc
Gco3018	cgattccactgtggtttccaaaatcgtc
Gco3040	atattgatttatttcgagttgcttgagaaa
Gco3070	gctacttatttcacgaccaagctgcggaac
Gco3071	tgcaagctgacagcaagttctacga
Gco3072	gacgcgatgaccggaaatatgccagttc
Gco3073	agcttttctctcacctccaacgcgagaa
Gco3081	gctccgatgtgatcctatagtgaatata
Gco3147	ctttctcgcgcctttgtctctctatcgg
Gco3148	gggacattttgcaacagagctcacacac
Gco3149	gagaggttcgattgagttcggggactga
Gco3202	tgaagaccttacgacggcac
GCo3223	cggtggaattcctcaacatc
GCo3224	ggatttgcttcaaagaccca
